# Unlocking high-intensity performance thresholds through ventilatory signatures in the ECG

**DOI:** 10.1038/s41598-026-53483-7

**Published:** 2026-05-19

**Authors:** V. Heinz, N. Pilz, L. Fesseler, T. Lindner, L. Malotka, O. Opatz, D. Blottner, O. Anosov, A. Patzak, Michael Fähling, T. L. Bothe

**Affiliations:** 1 Institute of Integrative NeuroAnatomy, Charité - University Medicine Berlin, Berlin, Germany; 2 Institute of Physiology, Hannover Medical School, Hanover, Germany; 3 Department of Cardiology and Angiology, Hannover Medical School, Hanover, Germany; 4 Institute of Translational Physiology, Charité - University Medicine Berlin, Berlin, Germany; 5 Department of Anesthesiology and Intensive Care Medicine, Charité - University Medicine Berlin, Berlin, Germany; 6 Menzies Institute for Medical Research, University of Tasmania, Hobart, Australia; 7 School of Health Sciences, The University of Sydney, Sydney, Australia; 8 Institute of Sport Science, Leibnitz University Hannover, Hannover, Germany

**Keywords:** Ventilatory threshold, Performance thresholds, ECG-derived respiration, Cardiopulmonary exercise testing, Exercise intensity assessment, Cardiology, Health care, Medical research, Physiology

## Abstract

A ubiquitously available and accurate non-invasive ventilatory threshold assessment (NIVA) would substantially improve real-world performance assessment evaluation in both clinical and elite sports settings. We hypothesised that ECG-derived ventilatory phase analysis achieves reference-standard accuracy for second ventilatory threshold (VT2) determination. 74 healthy adults performed stepwise cardiopulmonary exercise testing with simultaneous lactate sampling to retrieve VT2 and lactate-based (Dmax; LT2) thresholds. Threshold agreement was evaluated for heart rate (HR) and exercise load (W) between VT2, LT2, age-estimated HR (HR-Est) and NIVA. In 66 assessable datasets, NIVA and VT2 yielded equivalent threshold estimates for HR (− 0.46 bpm; 90% CI [− 2.10;1.17]) and exercise load (0.46 W; 90% CI [− 2.35; 3.27]). VT2 and HR-Est diverged (HR − 7.22 bpm, *p* < 0.001; load − 6.26 W; *p* < 0.001). LT2 was available in 58 subjects and differed from both VT2 (*p* < 0.001) and NIVA (*p* < 0.001). Correlations supported these findings, with close associations between VT2 and NIVA (HR *r* = 0.84; load *r* = 0.96). NIVA derived a high-intensity performance threshold from ECG signals with reference-standard fidelity and showed close agreement with CPET-derived VT2. Its performance and accessibility make it attractive for frequent reassessment of a VT2-aligned threshold without the need for spiroergometry or lactate measurements. Validation across devices, protocols, populations, and real-world signal conditions is warranted.

## Introduction

The second ventilatory threshold (VT2) and the second lactate threshold (LT2) are well-established markers for quantitative intensity steering in endurance training as well as for the assessment of overall cardiorespiratory fitness^1^. They reflect the transition to exercise intensities at which lactate production exceeds clearance, resulting in progressive blood lactate accumulation and a subsequent increase of ventilation, and carbon dioxide elimination^2^.

The VT2/LT2 are associated with related conceptual frameworks describing the upper boundary of sustainable exercise intensity, including critical power, maximal lactate steady state, and maximal metabolic steady state. Although these constructs differ methodologically and are not interchangeable, they aim to characterise a comparable physiological performance threshold marking the transition from steady-state to non-steady-state exercise domains^3–5^. Exercise sustained above this transition is characterised by metabolic instability and limited tolerability, with implications for fatigue development with an increased risk of overtraining, risk of injury, and post-exercise discomfort depending on population and training context^6,7^. Conversely, predominantly low-intensity distribution may attenuate performance improvements^6,8^. A clear and operational definition of VT2/LT2 remains central to both clinical exercise testing and performance practice^9,10^.

Routine clinical and athlete testing protocols involve the assessment of the performance threshold (VT2 during cardiopulmonary exercise testing (CPET)) and by lactate-based criteria, both performed during stepwise or ramp protocols^10^. CPET assesses ventilatory thresholds by analysing breath-by-breath oxygen uptake (V̇O₂) and carbon dioxide output (V̇CO₂). Performance thresholds can be determined using the V-Slope approach^11–13^. Lactate-based assessments require capillary blood sampling during incremental loading and curve-based analysis (e.g. Dmax-method)^14,15^. Both approaches are widely used reference standards for threshold detection and are routinely applied in high-performance sport and clinical decision-making^9,10,14^.They enable graded exercise prescription across a spectrum from rehabilitation to elite athlete conditioning^1,16^.

Despite their strengths, the gold standard methods face practical constraints, which limit scalability and real-world applicability. They require a controlled laboratory environment, specialised equipment, and trained personnel. Both approaches are resource-intensive, locally constrained, and impractical for frequent reassessment^2,9^. Protocol selection, execution, and inter-rater variability can also shift the derived threshold, even under optimised conditions^17^. Moreover, lactate testing is invasive, can be uncomfortable and carries the risk of localized infections. These barriers matter in real-world settings, from rehabilitation settings where cost is paramount to elite sport where adaptability and flexibility are crucial^9^.

A common, more practical option is heart-rate-based estimation (HR-Est), typically via fixed percentage of age-predicted maximal heart rate (HRmax) or heart rate reserve^18^. It is integrated within most consumer wearable sport devices and therefore naturally widely adopted, especially in the large group of recreational athletes^19,20^. However, accuracy varies across populations and between individuals as well as being dependent on the exact exercise protocol, meaningfully limiting the reliability of HR-Est surrogates for VT2^18,21^.

Heart-rate-variability (HRV) derived thresholds offer an alternative non-invasive approach^22–24^. However, agreement with gold-standard methods varied considerably with protocol and population, complicating standardisation and broad implementation^25^. Moreover, in some individuals and populations, HRV methods fail entirely to identify thresholds or produce inconsistent results^26,27^. HRV-based approaches remain attractive, but heterogeneity across methods has limited consistent uptake beyond controlled research settings^25,28,29^.

A more direct path could be to exploit the respiratory rhythm which is modulated by crossing the VT2/LT2 following physiological signal monitoring. As increased lactate production increases ventilation to reduce blood CO_2_ and adjust the pH, characteristic changes in the breathing pattern appear and can be captured^2,13,30^. Previous studies using wearable respiratory sensors showed promising agreement with ventilatory thresholds at the population level, but individual accuracy remained to be established^31^. Importantly, respiratory information can also be extracted from a standard electrocardiogram (ECG), offering a signal approach already present in most clinical and many performance contexts^32,33^. Early studies explored the potential improvement of threshold detection using respiration-related signal dynamics, including breakpoint analysis of breathing rate information. This approach showed substantial improvements in pilot threshold detection from a non-invasive and easily attainable signal, which could enable the potential of frequent reassessment^34,35^. However, existing evidence is limited to ramp-cycling protocols and cohorts that do not span the full fitness spectrum. As a result, generalisability to diverse real-world settings and step-increment testing remains uncertain^30,31^. We hypothesised our refined approach of non-invasive ventilatory threshold assessment (NIVA) could provide accurate determination in a wide range of fitness levels and when performed during a classical bike ergometer step-increase load profile.

In this study, we evaluate NIVA based on ECG-derived breathing phase information during incremental exercise to determine the individual performance threshold. We assessed whether NIVA yields heart rate and exercise load values equivalent to the VT2 and its accuracy difference related to routine HR-Est and lactate-derived thresholds.

## Methods

### Participants

A total of 74 healthy participants with varying levels of fitness (recreational to professional athletes) were included. Prior to enrolment, a resting ECG was recorded, and only those with a normal result were eligible. Participants were instructed to refrain from eating for at least two hours prior to testing, with only water permitted. They were specifically asked to avoid caffeine and nicotine during this period. Additionally, all participants were requested to abstain from physical exercise for 24 h prior to testing. Exclusion criteria comprised medication intake, abnormal ECG findings or pre-existing medical conditions.

### Cardiopulmonary exercise testing

All participants performed a stepwise incremental CPET on a bicycle ergometer (Lode Corival 906900, Lode B.V., Groningen, Netherlands) with simultaneous spirometry assessment (CORTEX MetaLyzer 3B, Leipzig, Germany). The ambient room temperature was maintained at 23 °C by air conditioning. The initial workload was adjusted individually, starting at 50 W for females and 80 W for males. The workload was increased by 30 W every 3 min. Participants were instructed to maintain the cadence at 70 rpm. Termination criteria included volitional exhaustion or when subjects reached their maximum heart rate (calculated as 220 minus age in years)^36^.

### Electrocardiography

Continuous three-lead ECG monitoring with the CustoGuard Holter (custo med, Ottobrunn, Germany) was performed during CPET to retrieve the heart rate (HR).

### Ventilatory and lactate threshold determination

#### Respiratory gas analysis

The VT2 was determined through respiratory gas analysis using the V-Slope method^13^. The detected threshold was validated through blinded dual-investigator inspection to ensure accuracy. In the case of investigator disagreement of more than 30 s, a third investigator was asked to resolve the disagreement.

#### Capillary blood gas analysis

The LT2 was assessed via capillary blood samples collected from the right earlobe using the Dmax method^14^. Samples were taken at baseline as well as after 2 min in each load interval, and 2 min post-exhaustion. Blood lactate levels were analysed using a blood gas analyser (BGA; ABL800 Plus, ABL835 Version 6.20 MR1, Radiometer GmbH, Krefeld, Germany)^37^. The BGA underwent daily calibration according to the manufacturer guidelines. LT2 was derived with the Dmax method^38^.

### Heart rate-based threshold estimation

HR-Est was defined as 85% of age-predicted maximal heart rate, using HRmax = 220 − age; thus, HR-Est = 0.85 × (220 − age)^39^.

### Non-invasive ventilatory threshold assessment

NIVA is a signal-processing and sequence-modelling framework designed to identify the high-intensity ventilatory transition (corresponding to VT2/LT2) by analysing ventilatory phase dynamics derived from physiological signals.

In this study, respiratory information was extracted from single-channel ECG recordings obtained during exercise at a sampling frequency of 256 Hz. Lead II was used for analysis in all participants except one, in whom lead III was analysed due to loss of the lead II signal (electrode detachment). Raw ECG recordings were processed to extract beat-to-beat RR intervals. The resulting RR time series was subjected to band-limited frequency filtering to isolate respiratory-related oscillatory components while suppressing non-respiratory variability, by using a band-pass filter in the frequency domain of breathing patterns during exercise. A modified Fourier-based multiplier transformation was then applied to enhance phase-deflection characteristics associated with ventilatory modulation during incremental exercise. This results in a semi-continuous respiratory phase trace. NIVA detects a characteristic, non-continuous feature within this phase-deflection signal which corresponds to VT2/LT2.

The framework encompasses, in addition to the mathematical steps, two sequential long short-term memory (LSTM) modules. The first LSTM performs RR interval extraction from the raw ECG signal. The R-peak detection model consisted of four stacked bidirectional LSTM (biLSTM) layers with h1 to h4 hidden units per direction. Input segments comprised multiple seconds and were zero-padded where needed. The final biLSTM output was passed to a dense layer with u1 units and a sigmoid output layer for sample-wise prediction. To reduce physiologically implausible detections, model predictions were post-processed using a refractory-period constraint of 250ms. If multiple candidate peaks occurred within this window, only the peak with the highest prediction score was retained. The second LSTM analyses the resulting latent temporal phase representation to identify characteristic inflection behaviour within the respiratory-modulated signal. A four-layer LSTM architecture was used to detect a single breakpoint in continuous signals. Prior to model input, all signals were resampled to 1 Hz. To accommodate variable recording duration in the production setting, sequences were zero-padded only as required up to a maximum supported length of 1 h, and padded regions were excluded from computation by masking. The sequences were processed by four stacked LSTM layers with h1–h4 hidden units, and the final hidden representation was mapped to a dense output layer. The output layer generated a probability distribution over all time points in the sequence, and the breakpoint was defined as the time point with the maximum predicted probability, thereby enforcing a single estimate per recording. The model outputs a single time-point corresponding to the inferred ventilatory transition, which is subsequently mapped to heart rate and exercise load values.

The machine-learning architectures and the complete NIVA algorithm were developed prior to this present investigation, based on an independent ECG dataset obtained in previous internal studies conducted by the research group. In total, 50 anonymised cardiopulmonary exercise tests were used, with VT2 determined from the 50/30/3 protocol. The dataset comprised healthy recreational-to-athletic adults aged 18–40 years (26.8 ± 6.2 years, BMI 23.6 ± 2.5, Maximum W 193.1 ± 51.6, 50% women), representing a broad fitness spectrum. This dataset was fully independent of the validation dataset discussed in this paper.

No retraining, parameter tuning, or model adaptation was performed using the current study cohort. The present study therefore represents an external physiological validation of a pre-specified and fixed model.

The underlying signal-processing framework is subject to an international patent application (PCT/EP2025/088723). The conceptual processing steps are disclosed here to ensure scientific transparency, while specific implementation parameters remain protected under the ongoing intellectual property procedure.

### Data analysis and statistics

Statistical analyses and graphical representations were performed using Python (version 3.13.2). HR and exercise load at the respective performance thresholds (VT2, LT2, HR-Est, and NIVA) were compared across methods. The primary comparison was performed between VT2, HR-Est, and NIVA. In all subjects with available LT2, we conducted a secondary comparison between VT2, LT2, and NIVA. To assess differences between the threshold detection methods, a Bonferroni corrected repeated measures ANOVA with a post-hoc paired T-test was applied. In case of no differences in the ANOVA analyses, we carried out subsequent two one-sided tests (TOST) to test for equivalence with provided 90% confidence intervals (CI). Predefined equivalence margins were set at ± 5 bpm for HR and ± 5 W for exercise load. These thresholds were chosen as conservative allowances for physiological heart-rate variability, including respiratory modulation, and for the limited precision of workload control under real-world conditions. Agreement between the threshold determination methods was evaluated using linear regression and Bland-Altman analyses. In addition, Steiger’s Z test was applied to compare the strength of correlations. The alpha error threshold was set at *p* < 0.05.

In correspondence with the Charité’s institute of biometrics, sample size estimation was performed based on correlation coefficients. Assuming an expected correlation of *r* = 0.85, 63 evaluable participants yield an approximate 95% confidence interval of 0.76 to 0.91. Allowing for an anticipated dropout rate of 15%, the target sample size was set at 74 participants.

### Ethics

Ethical approvalfor this study was granted by the Ethics Committee of Charité–Universitätsmedizin Berlin (approval number: EA2/165/23). The study was conducted in accordance with the Declaration of Helsinki and its later amendments as well as under the guidance of European and Federal German law^40^. Before participation, all subjects provided written informed consent.

### Equity, diversity, and inclusion statement

Participants were adults of different ages and both sexes. Recruitment did not apply restrictions based on protected characteristics, and all procedures and analyses were applied uniformly.

## Results

### Participant characteristics

A total of 74 participants (38 male, 36 female) were enrolled in the study. Eight individuals were excluded from analysis due to ECG artifacts (five cases) or inter-rater disagreement in VT2 determination (three cases). The final analytical cohort for the primary VT2, HR-Est, and NIVA comparison comprised 66 participants (35 male, 31 female). For the secondary VT2, LT2, and NIVA comparison, another eight individuals were excluded from analysis due to missing lactate data or inapplicability of the Dmax method, resulting in 58 (31 male, 27 female) analysed datasets. (Table 1) For all comparisons there was no systematic sex difference observable in our data.

**Table 1:** Anthropometric and physiological characteristics of participants included in the primary and secondary analysis. Values are presented as mean ± standard deviation with range (minimum–maximum) in parentheses. BMI = body mass index; VT = ventilatory threshold expressed in W/kg.

Participants characteristics - Primary comparison (*n* = 66)			
Parameter	Total	Male	Female
Age (years)	28.95 ± 4.73 (20–43)	29.06 ± 4.75 (20–43)	28.84 ± 4.79 (20–43)
Height (cm)	176.4 ± 8.5 (157–197)	181.5 ± 6.2 (173–197)	170.6 ± 6.9 (157–185)
Weight (kg)	72.3 ± 12.1 (48–105)	80.3 ± 9.5 (65–105)	63.3 ± 7.5 (48–79)
BMI (kg/m²)	23.11 ± 2.51 (16.8–30.0)	24.34 ± 2.29 (20.8–30.0)	21.72 ± 1.98 (16.8–25.5)
VT2 (W/kg)	2.70 ± 0.51 (1.69–4.34)	2.77 ± 0.60 (1.69–4.34)	2.62 ± 0.40 (1.90–3.26)
Participant characteristics – Secondary comparison (*n* = 58)			
Age (years)	28.84 ± 4.79 (21–43)	28.94 ± 4.75 (20–43)	28.74 ± 4.93 (21–39)
Height (cm)	177.0 ± 8.4 (157–197)	182.0 ± 6.3 (174–197)	171.3 ± 6.7 (162–185)
Weight (kg)	72.9 ± 11.9 (48–105)	80.7 ± 9.4 (66–105)	64.0 ± 7.5 (48–79)
BMI (kg/m²)	23.15 ± 2.46 (16.8–30.0)	24.34 ± 2.24 (20.8–30.0)	21.78 ± 1.97 (16.8–25.5)
VT2 (W/kg)	2.77 ± 0.50 (1.90–4.34)	2.86 ± 0.56 (1.99–4.34)	2.66 ± 0.40 (1.90–3.26)

### Primary comparison between VT2, HR-Est, and NIVA

**Fig. 1:**
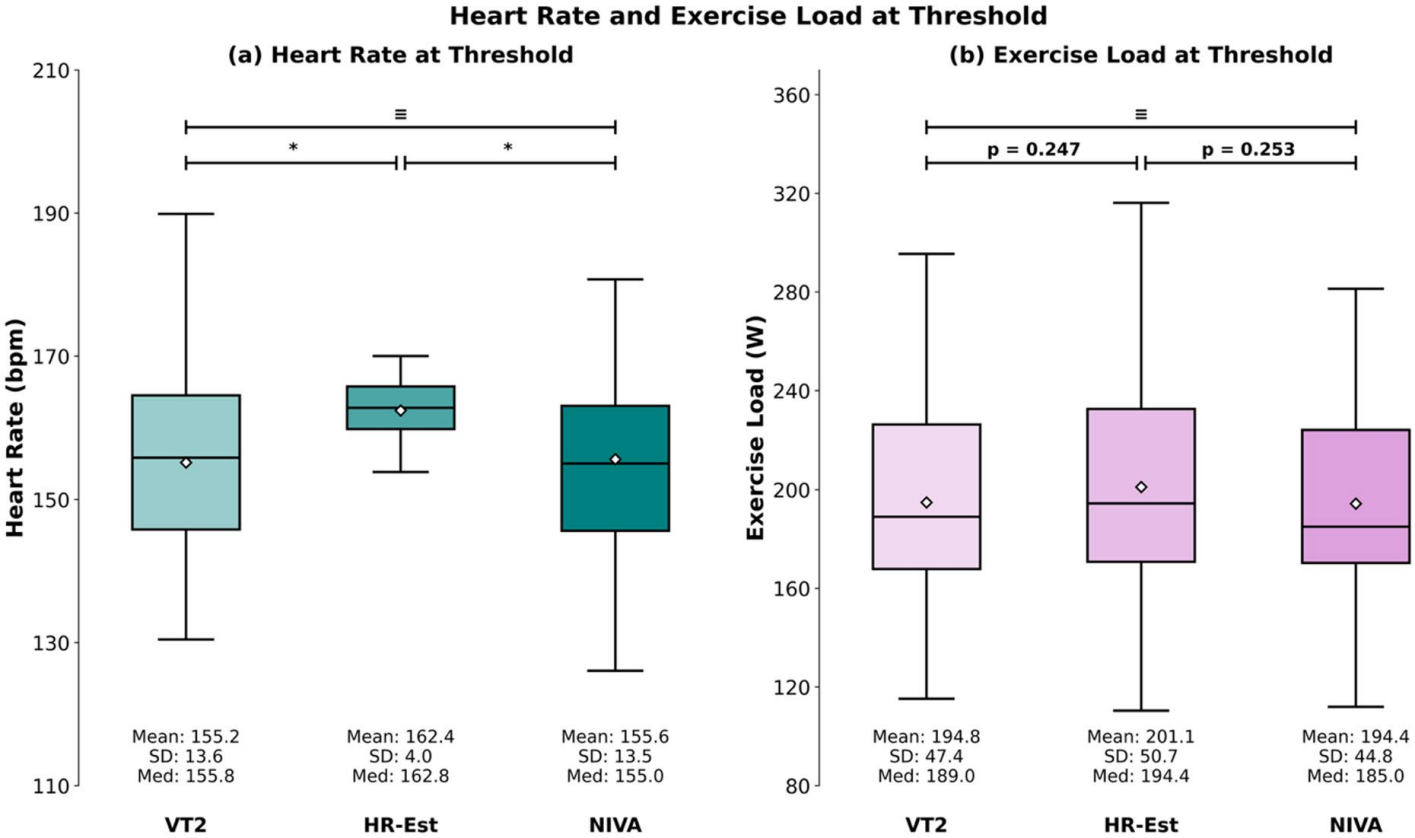
Panel (**a**) displays heart rate values determined at VT2, HR-Est, and NIVA. Panel (**b**) illustrates exercise load at threshold for VT2, HR-Est, and NIVA. Boxplots depict mean (diamond), median (horizontal line), the interquartile range (IQR, box) and the maximum value within 1.5 x IQR of the lower and upper quartile (whiskers). * = *p* < 0.05; ≡ = statistical equivalence. VT2 = Second Ventilatory Threshold. HR-Est = Heart rate estimation, NIVA = non-invasive ventilatory threshold assessment.

For *n* = 66 subjects, the HR thresholds, comparison between VT2, NIVA, and HR-Est retrieved group differences in the ANOVA (*p* < 0.001). Post-hoc testing highlighted that HR-Est provided higher HR values than both VT2 (mean difference = -7.22; SD = 4.0; *p* < 0.001) and NIVA (mean difference = -6.75; SD = 13.27; *p* < 0.001). Equivalence testing between VT2 and NIVA confirmed equivalence (*p* < 0.001) and thereby retrieved a 90% CI within the predefined equivalence margin of ± 5 bpm [-2.10; 1.17 bpm]. (Fig. 1)

**Fig. 2:**
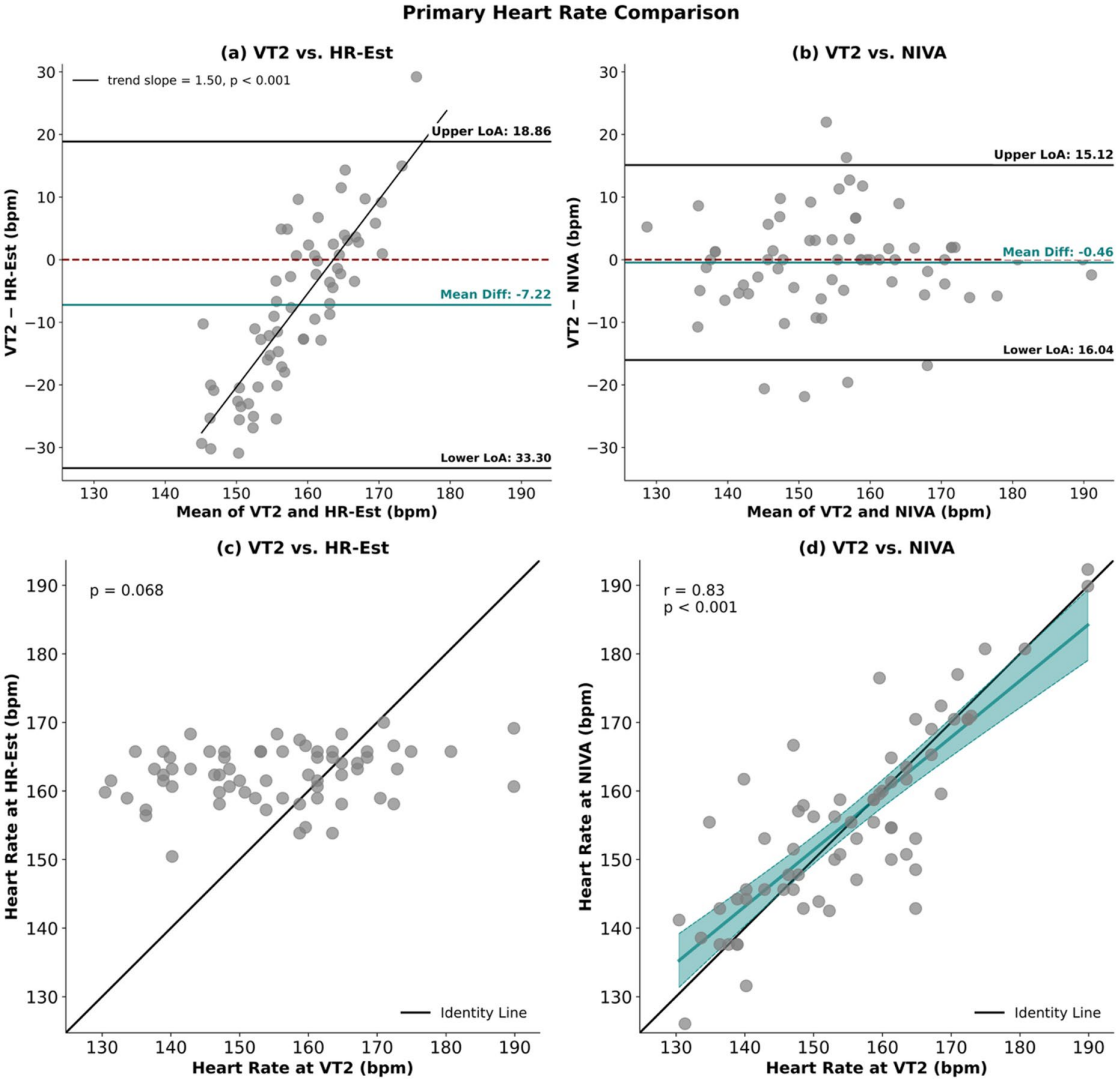
Panels (a) and (b) display Bland–Altman plots between VT2 and HR-Est (a) and VT2 and NIVA (b). The differences between methods (VT2 – comparator) are plotted against their mean values. The mean bias is shown as a central teal line, the dashed red line indicates the reference of zero mean difference, and the black horizontal lines represent the upper and lower limits of agreement (LoA = bias ± 1.96 SD). Panels (c) and (d) illustrate the linear association between VT2 and HR-Est (c) as well as VT2 and NIVA (d). Data are shown as scatterplots with the line of identity (black) and, for panel (d), the fitted regression line including 95% confidence intervals (teal). Correlation coefficients (r) with corresponding p-values are provided.

Bland–Altman analysis between VT2 and HR-Est retrieved a mean bias of -7.22 bpm, with limits of agreement (LoA) ranging from − 33.30 to 18.86 bpm. For VT2 and NIVA, the mean bias was − 0.46 bpm, with LoA from − 16.0 to 15.1 bpm. (Fig. 2)

Correlation analyses showed an association between NIVA and VT2 (*r* = 0.83). (Fig. 2)

**Fig. 3:**
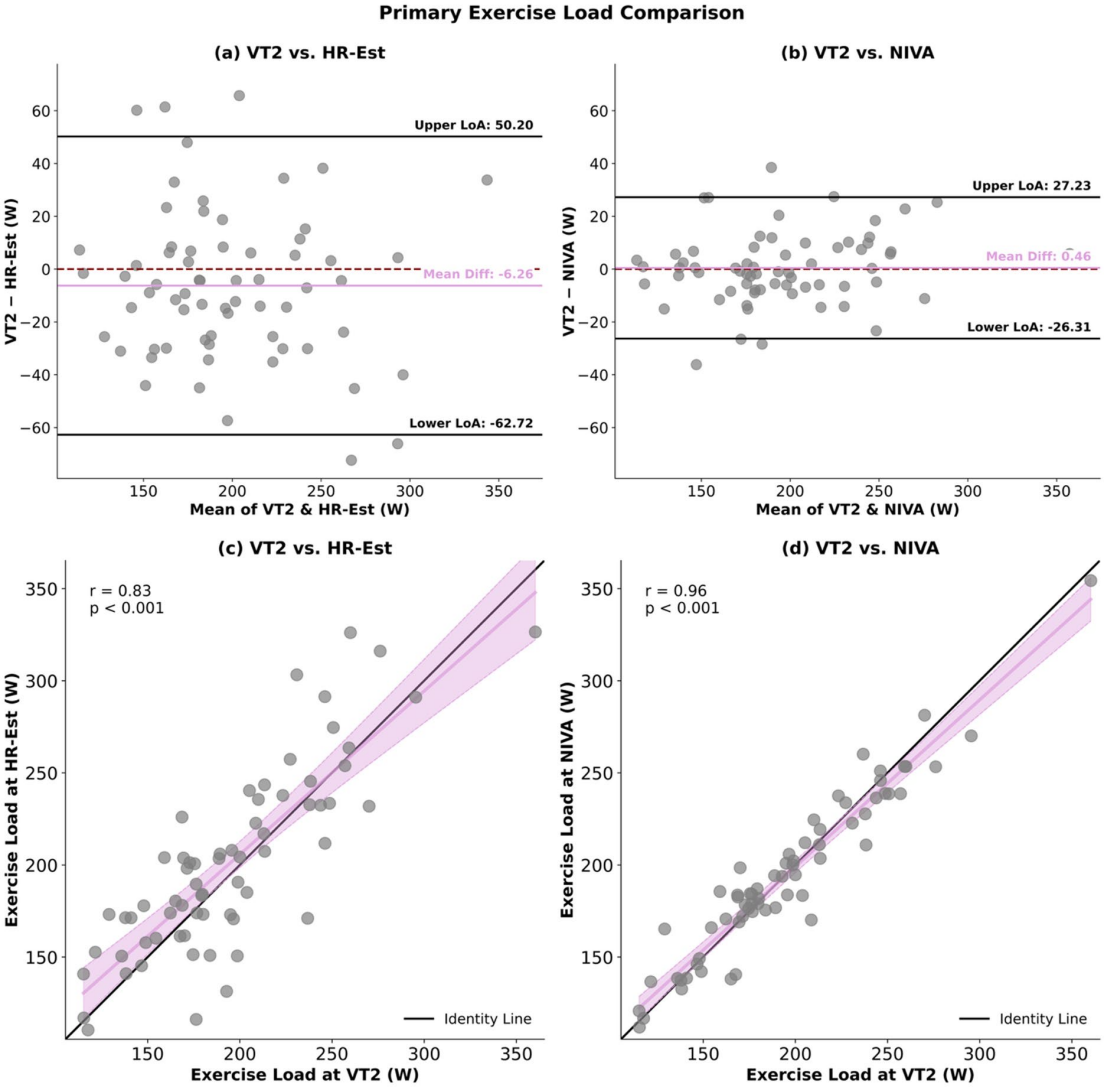
Panels (a) and (b) display Bland–Altman plots between VT2 and HR-Est (a) and VT2 and NIVA (b). The differences between methods (VT2 – comparator) are plotted against their mean values. The mean bias is shown as a central purple line, the dashed red line indicates the reference of zero mean difference, and the black horizontal lines represent the upper and lower limits of agreement (LoA = bias ± 1.96 SD). Panels (c) and (d) illustrate the linear association between VT2 and HR-Est (c) as well as VT2 and NIVA (d). Data are shown as scatterplots with the line of identity (black) and, for both panels, the fitted regression line including 95% confidence intervals (purple). Correlation coefficients (r) with corresponding p-values are provided.

For exercise load, no overall difference was observed (ANOVA, *p* > 0.05). Equivalence testing between VT2 and NIVA retrieved a 90% CI within the predefined equivalence margin of ± 5 W [− 2.35; 3.27 W]. Equivalence between VT2 and HR-Est could not be established, as the 90% CI [− 12.18; −0.34 W] exceeded the criterion. (Fig. 1) Bland–Altman analysis between VT2 and HR-Est retrieved a mean bias of − 6.26 W, with LoA ranging from − 62.72 to 50.20 W. For VT2 and NIVA, the mean bias was 0.46 W, with LoA from − 26.31 to 27.23 W. (Fig. 3)

Correlation analyses supported these findings and showed strong associations between VT2 and HR-Est (*r* = 0.83) and between VT2 and NIVA (*r* = 0.96). (Fig. 3)

### Secondary comparison between VT2, LT2, and NIVA

**Fig. 4:**
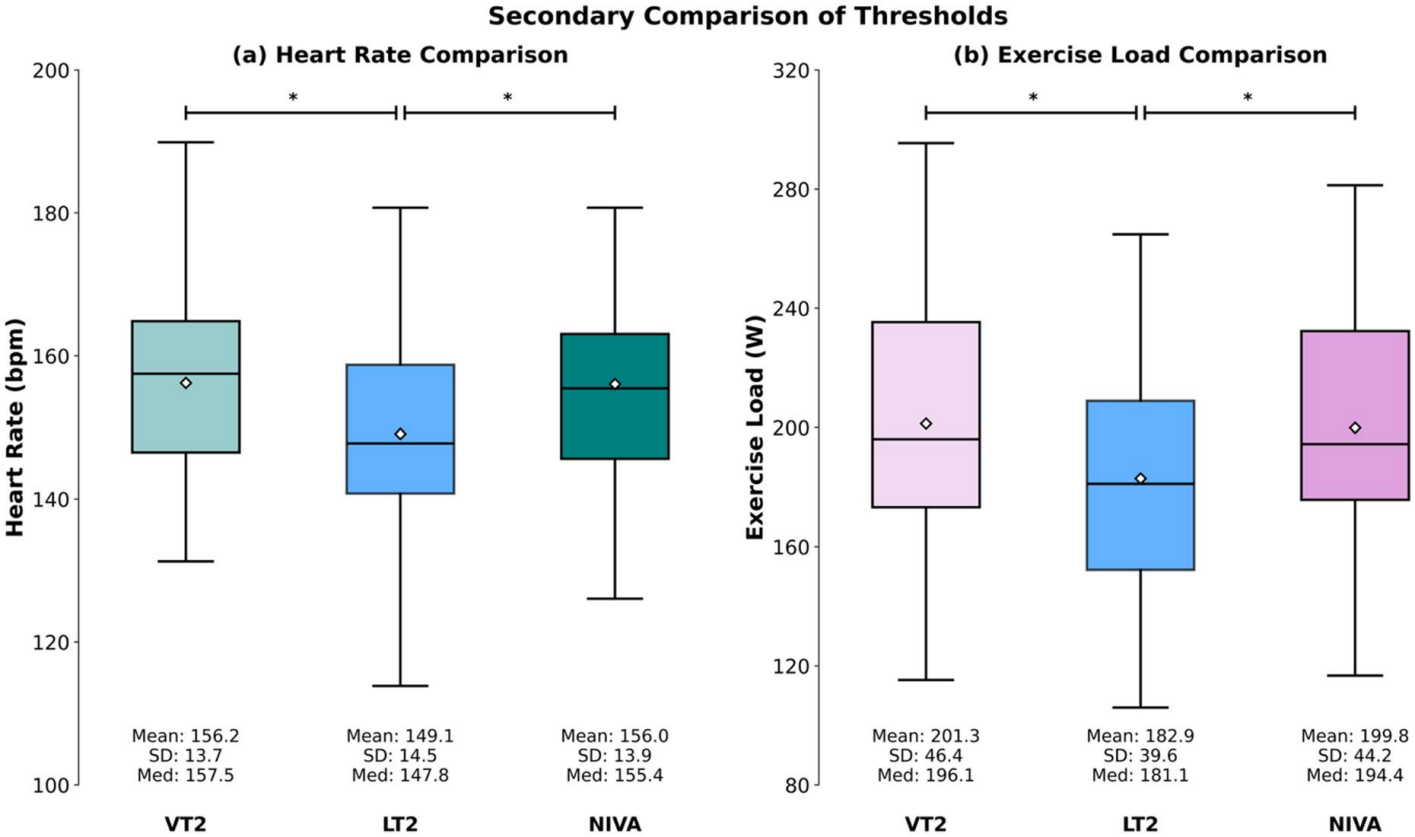
Panel (a) displays heart rate values determined at VT2, LT2, and NIVA. Panel (b) illustrates exercise load at threshold for VT2, LT2, and NIVA. Boxplots depict mean (diamond), median (horizontal line), the interquartile range (IQR, box) and the maximum value within 1.5 x IQR of the lower and upper quartile (whiskers). * = *p* < 0.05; ≡ = statistical equivalence. VT2 = Second Ventilatory Threshold. LT2 = Second Lactate Threshold, NIVA = non-invasive ventilatory threshold assessment.

For HR, the comparison of NIVA with VT2 and LT2 retrieved group differences in the ANOVA (*p* < 0.001). Post-hoc testing highlighted that LT2 provided lower HR values than both VT2 (*p* < 0.001) and NIVA (*p* < 0.001). (Fig. 4)

**Fig. 5:**
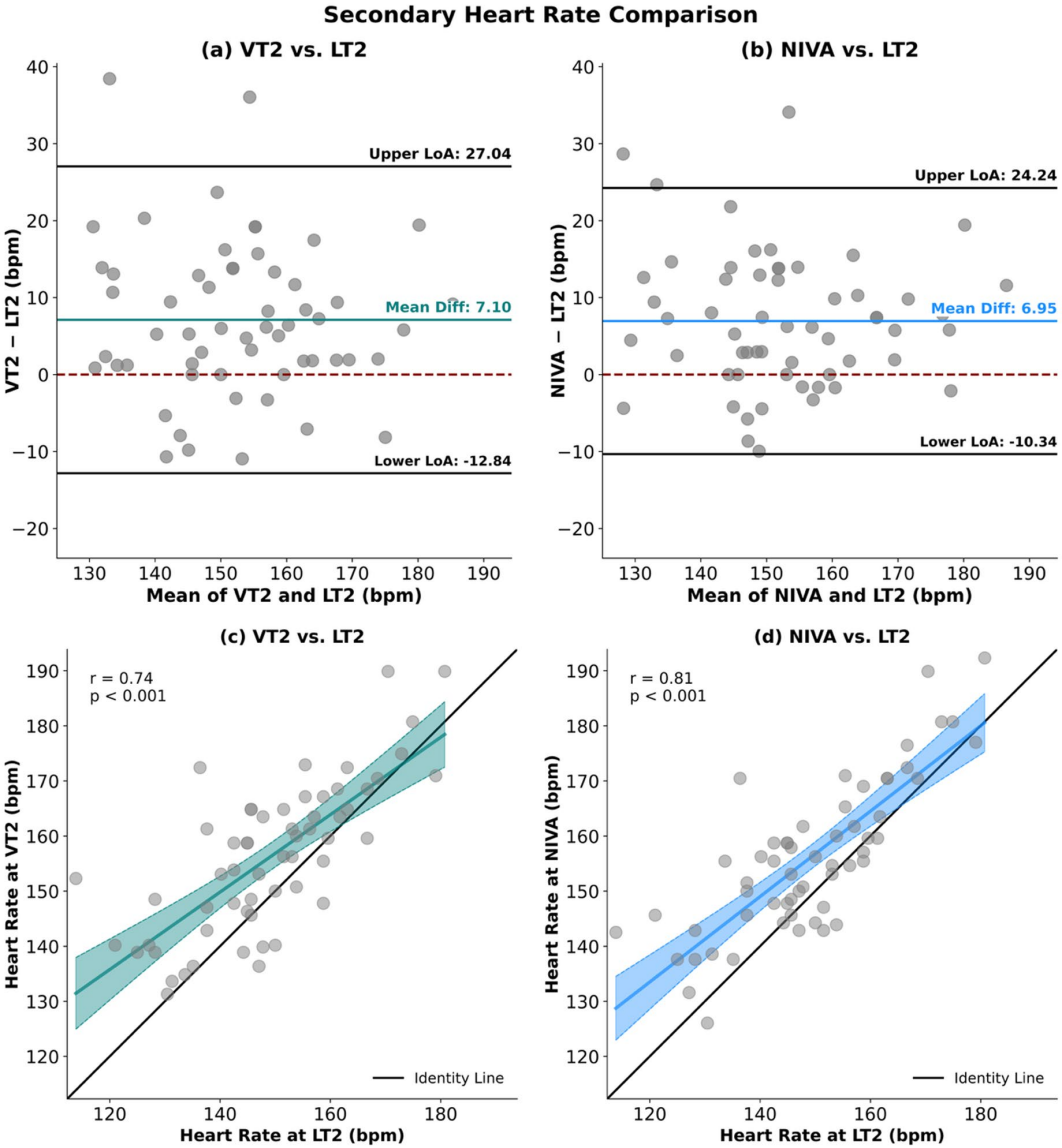
Panels (a) and (b) display Bland–Altman plots between VT2 and LT2 (a), and NIVA and LT2 (b). The differences between methods (VT2 – comparator or NIVA – LT2) are plotted against their mean values. The mean bias is shown as a central teal or blue line, the dashed red line indicates the reference of zero mean difference, and the black horizontal lines represent the upper and lower limits of agreement (LoA = bias ± 1.96 SD). Panels (c) and (d) illustrate the linear association between VT2 and LT2 (c), and LT2 and NIVA (d). Data are shown as scatterplots with the line of identity (black) and the fitted regression line including 95% confidence intervals (green for panels c, blue for panel d). Correlation coefficients (r) with corresponding p-values are provided.

Bland–Altman analysis between VT2 and LT2 retrieved a mean bias of 7.10 bpm, with LoA ranging from − 12.84 to 27.04 bpm. For LT2 and NIVA, the mean bias was 6.95 bpm, with LoA from − 10.34 to 24.24 bpm. (Fig. 5)

Correlation analyses showed associations between VT2 and LT2 (*r* = 0.74), VT2 and NIVA (*r* = 0.84), and LT2 and NIVA (*r* = 0.81). (Fig. 5)

For exercise load, ANOVA likewise indicated group differences (*p* < 0.001). Post-hoc comparisons demonstrated that LT2 yielded lower values compared to both VT2 (*p* < 0.001) and NIVA (*p* < 0.001). Bland–Altman analysis between VT2 and LT2 retrieved a mean bias of 18.36 W, with LoA ranging from − 20.91 to 57.64 W. For LT2 and NIVA, the mean bias was 16.94 W, with LoA from − 16.42 to 50.31 W.

**Fig. 6:**
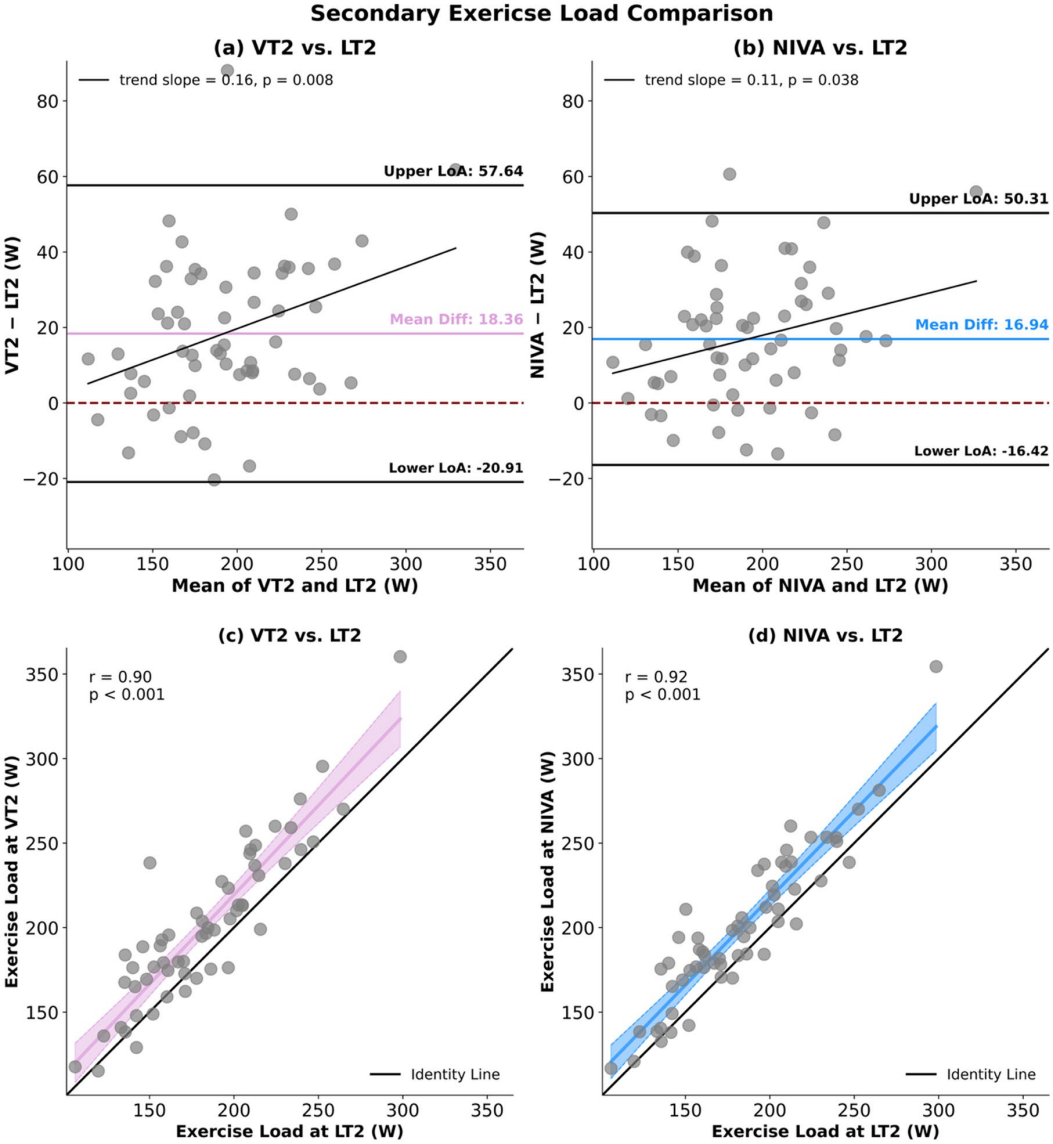
Panels (a) and (b) display Bland–Altman plots between VT2 and LT2 (a), and LT2 and NIVA (b). The differences between methods (VT2 – comparator or NIVA – LT2) are plotted against their mean values. The mean bias is shown as a central purple or blue line, the dashed red line indicates the reference of zero mean difference, and the black horizontal lines represent the upper and lower limits of agreement (LoA = bias ± 1.96 SD). Panels (c) and (d) illustrate the linear association between VT2 and LT2 (c), and LT2 and NIVA (d). Data are shown as scatterplots with the line of identity (black) and the fitted regression line including 95% confidence intervals (purple for panels c, blue for panel d). Correlation coefficients (r) with corresponding p-values are provided.

Correlation analyses showed strong association between VT2 and LT2 (*r* = 0.90), and between LT2 and NIVA (*r* = 0.92). (Fig. 6)

## Discussion

We systematically evaluated a novel method to determine the individual performance threshold from respiratory phase deflection signals derived from ECG during a standard bike ergometer incremental exercise test. We compared the approach against established gold-standard spiroergometry and a lactate method. Consistent with our a priori equivalence margins, NIVA and VT2 were equivalent for both heart rate and exercise load (± 5 bpm; ±5 W), with 90% CIs fully contained within these bounds. By contrast, there was a difference between VT2 and LT2, and between VT2 and HR-Est. Correlation analyses were consistent with the equivalence and agreement findings and further supporting its positioning as a methodologically aligned surrogate of VT2. Taken together, these findings validate that NIVA can retrieve the VT2 with laboratory-comparable fidelity within a pragmatic ± 5 bpm / ±5 W boundary.

As exercise progresses from moderate to heavy and then to severe intensities, lactate accumulation increases the hydrogen ion load. Bicarbonate buffering of these protons generates additional carbon dioxide, which also stimulates chemoreceptors and drives a disproportionate rise in ventilation, accompanied by characteristic changes in breathing pattern and timing^14,41^. When thresholds are identified from gas exchange, they are anchored in this respiratory response, reflecting the transient change in the metabolic state. Because NIVA interprets respiratory behaviour extracted from raw ECG signals, its absolute estimates would be expected to align closely with VT2. In contrast, LT2, which samples lactate concentrations whose increase is a chemical marker for the metabolic shift, introduces additional detection (measurement fidelity) and modelling (curve fit through sparse data points) variability. This interpretation is in line with contemporary clinical guidance, which favours prescription based on ventilatory thresholds^9,10,31^. Compared with consumer-grade ventilatory threshold estimation, our study revealed clear differences between HR-Est thresholds and VT2. In contrast, NIVA showed equivalence (negligible) mean difference, limits of agreement narrower than those reported for VT2 versus LT2, and variability within the range inherent to spirometric threshold determination, extending the very strong correlation results^42^. This observation aligns with current literature, as HR–based threshold estimation using fixed percentage rules often fails to reflect individual ventilatory thresholds, leading to misclassification of training intensity, particularly in cardiac patients and women^18,43–45^.

Looking beyond the scope of this study, HRV derived thresholds continue to attract attention^10^. However, substantial inter-individual variability and methodological inconsistency limit their standalone utility^22,25,46^. Recently, a sweat-based lactate sensor estimated the thresholds in heart-failure patients with good agreement to the ventilatory threshold, underscoring the clinical interest in non-invasive assessment methods^47^. Nevertheless, these technologies lack systematic validation across a wide range of performance capability^48,49^. Developing a ventilatory threshold detection approach that combines reference-standard accuracy with field practicality could substantially advance performance diagnostics in both clinical and athletic domains. The NIVA approach validated in this study does not require a highly specific load increase profile but showed surprising accuracy even within a classic stepwise increase profile. Further, the breathing phase deflection approach is potentially transferable into real-world and even outdoor performance testing across a wide range of sports (e.g., running, cycling, rowing, etc.). It could be facilitated using various sensors (e.g., HR chest-belt) or even different sensor modalities (e.g., breathing belt, chest-worn accelerometer, etc.)^33^. Further validation in these scenarios and with a range of devices and modalities is warranted.

Several limitations should be acknowledged. Determining ventilatory thresholds requires expertise and remains partly observer dependent, adding variability^9,50,51^. Recent studies showed that agreement on key CPET variables improves with guideline-based systematic reading. However, inter-observer variability and inherent, relevant methodological limitations persist, especially for threshold identification. The standard deviation between observers in CPET-derived VT2 estimation has been reported to be approximately ± 8% for experienced physicians^52^. Applied to our dataset, this corresponds to a heart rate standard deviation of ± 12.4 bpm and an exercise load standard deviation of ± 15.6 W. The defined equivalence margins of ± 5 bpm and ± 5 W, representing the minimal steerable exercise intensity, are more than 50% lower than the expected inter-observer variability and therefore constitute a conservative threshold for agreement. This interpretation is further supported by prior work on ventilatory threshold reliability and methodological variability in CPET interpretation^42,50–53^. In addition, inter-observer variability in CPET-derived VT2 determination has been reported with an intraclass correlation coefficient of 0.83 (0.75–0.90)^17^. Based on the distribution of our dataset, this corresponds to a minimal detectable change at the 95% confidence level (MDC95) of 15.5 bpm for heart rate and 54.2 W for workload. As the MDC95 reflects the expected variability between repeated or independent assessments, it provides a direct reference for interpreting the Bland–Altman limits of agreement. In this context, the observed limits of agreement between NIVA and VT2 are comparable for heart rate (± 15–16 bpm) and smaller for workload (± 26–27 W) than the expected inter-observer variability of CPET-derived VT2. In practical terms, the observed limits of agreement suggest that NIVA may affect fine-grained individual prescriptions near zone boundaries but are unlikely to alter broader physiological intensity-domain classification in most cases, which is relevant for athlete training-zone allocation and clinical exercise prescription. This challenge underscores the need for standardised procedures when using these measures for validation^50,51,53,54^. Additionally, our study was conducted in young and healthy adults, so caution should be applied before extrapolating to older, clinical or extreme fitness populations. We mitigated these issues by harmonising lactate sampling, consistent criteria for VT determination, and a blinded dual-investigator approach with a third rater acting as judge. We followed a step-increment cycling protocol to reflect the intended translation to field testing, where truly linear ramps are difficult to implement. A potential limitation of step protocols is that abrupt workload transitions may elicit transient cardiorespiratory responses. However, stepwise incremental protocols are established in CPET practice and remain commonly used alongside ramp approaches and steady-state cardiorespiratory responses typically require several minutes at a given constant workload^55,56^. Nevertheless, direct head-to-head comparisons between ramp and step protocols would be valuable to further examine protocol-related effects. Despite the different workload progression, our NIVA results were comparable to reports based on ramp protocols^31,34^. Furthermore, ECG signal quality posed practical challenges using a Holter ECG. In our cohort, 5 out of 74 participants (7%) were excluded due to artefacts or equipment malfunction despite standardised testing procedures. Real-world deployment will likely involve lower-fidelity and noisier signals (e.g., single-lead wearables). Although wearables (e.g., chest-strap systems) represent a simpler and potentially suitable alternative, especially considering the R-peak dependent approach of NIVA, their performance for this specific application remains to be established^33,57^. Accordingly, NIVA should be evaluated across devices, signal qualities, and protocol types, with prospective validation in naturalistic settings.

## Conclusion

NIVA provides a practical route to frequent and individualised performance ventilatory threshold assessment from ECG signals that are already widespread in exercise testing. In our study, NIVA’s threshold determination was equivalent to the laboratory VT2, confirming a level of accuracy comparable to CPET-derived ventilatory threshold assessment. If validated across diverse sports, protocols, and devices, and tested with the signal noise typical of field conditions, NIVA could offer VT2-aligned threshold assessment comparable to CPET while reducing complexity and resource demands for both clinicians and practitioners. Moreover, the ability to perform high-frequency measurements may provide deeper insights into temporal changes of performance thresholds and help to further identify potential influencing factors. Future studies should evaluate generalisability across sex, age, disease states and performance levels, benchmark its performance when used with consumer-grade devices, and explore integration with longitudinal monitoring to support training prescription, rehabilitation and risk screening.

## Data Availability

Due to an ongoing patent application covering key analytical components of the method, the full dataset and algorithmic details cannot be made publicly available at this time. Data may be made available upon reasonable request to the corresponding author once the patenting process is completed and legal restrictions no longer apply.

## References

[1] Anselmi, F. et al. The importance of ventilatory thresholds to define aerobic exercise intensity in cardiac patients and healthy subjects. *Scand. J. Med. Sci. Sports*. **31** (9), 1796–1808 (2021). 34170582 10.1111/sms.14007 PMC8456830

[2] Poole, D. C., Rossiter, H. B., Brooks, G. A. & Gladden, L. B. The anaerobic threshold: 50 + years of controversy. *J. Physiol.* **599** (3), 737–767 (2021). 33112439 10.1113/JP279963

[3] Murphy, C., Svansdottir, S. A., Dupuy, O. & Louis, J. Does overreaching from endurance-based training impair sleep: A systematic review and meta-analysis. *PLoS One*. **19** (5), e0303748 (2024). 38809828 10.1371/journal.pone.0303748 PMC11135706

[4] Carrard, J. et al. Diagnosing Overtraining Syndrome: A Scoping Review. *Sports Health*. **14** (5), 665–673 (2022). 34496702 10.1177/19417381211044739 PMC9460078

[5] Hill, D. W. The critical power concept. A review. *Sports Med.* **16** (4), 237–254 (1993). 8248682 10.2165/00007256-199316040-00003

[6] Silva Oliveira, P., Boppre, G. & Fonseca, H. Comparison of Polarized Versus Other Types of Endurance Training Intensity Distribution on Athletes’ Endurance Performance: A Systematic Review with Meta-analysis. *Sports Med.* **54** (8), 2071–2095 (2024). 38717713 10.1007/s40279-024-02034-z PMC11329428

[7] Soligard, T. et al. How much is too much? (Part 1) International Olympic Committee consensus statement on load in sport and risk of injury. *Br. J. Sports Med.* **50** (17), 1030–1041 (2016). 27535989 10.1136/bjsports-2016-096581

[8] Stöggl, T. & Sperlich, B. Polarized training has greater impact on key endurance variables than threshold, high intensity, or high volume training. *Front. Physiol.* **5**, 33 (2014). 24550842 10.3389/fphys.2014.00033 PMC3912323

[9] Glaab, T. & Taube, C. Practical guide to cardiopulmonary exercise testing in adults. *Respir Res.* **23** (1), 9 (2022). 35022059 10.1186/s12931-021-01895-6 PMC8754079

[10] Keir, D. A., Iannetta, D., Mattioni Maturana, F., Kowalchuk, J. M. & Murias, J. M. Identification of Non-Invasive Exercise Thresholds: Methods, Strategies, and an Online App. *Sports Med.* **52** (2), 237–255 (2022). 34694596 10.1007/s40279-021-01581-z

[11] Levett, D. Z. H. et al. Perioperative cardiopulmonary exercise testing (CPET): consensus clinical guidelines on indications, organization, conduct, and physiological interpretation. *Br. J. Anaesth.* **120** (3), 484–500 (2018). 29452805 10.1016/j.bja.2017.10.020

[12] Svedahl, K. & MacIntosh, B. R. Anaerobic threshold: the concept and methods of measurement. *Can. J. Appl. Physiol.* **28** (2), 299–323 (2003). 12825337 10.1139/h03-023

[13] Beaver, W. L., Wasserman, K. & Whipp, B. J. A new method for detecting anaerobic threshold by gas exchange. *J. Appl. Physiol.* **60** (6), 2020–2027 (1986). 3087938 10.1152/jappl.1986.60.6.2020

[14] Faude, O., Kindermann, W. & Meyer, T. Lactate threshold concepts: how valid are they? *Sports Med.* **39** (6), 469–490 (2009). 19453206 10.2165/00007256-200939060-00003

[15] Chalmers, S., Esterman, A., Eston, R. & Norton, K. Standardization of the Dmax method for calculating the second lactate threshold. *Int. J. Sports Physiol. Perform.* **10** (7), 921–926 (2015). 25710184 10.1123/ijspp.2014-0537

[16] Beneke, R., Leithäuser, R. M. & Ochentel, O. Blood lactate diagnostics in exercise testing and training. *Int. J. Sports Physiol. Perform.* **6** (1), 8–24 (2011). 21487146 10.1123/ijspp.6.1.8

[17] Abbott, T. E. F. et al. Inter-observer reliability of preoperative cardiopulmonary exercise test interpretation: a cross-sectional study. *Br. J. Anaesth.* **120** (3), 475–483 (2018). 29452804 10.1016/j.bja.2017.11.071

[18] Almaadawy, O., Uretsky, B. F., Krittanawong, C. & Birnbaum, Y. Target Heart Rate Formulas for Exercise Stress Testing: What Is the Evidence? *J. Clin. Med.* **13**, 18 (2024). 10.3390/jcm13185562 PMC11432587 39337046

[19] Fuller, D. et al. Reliability and Validity of Commercially Available Wearable Devices for Measuring Steps, Energy Expenditure, and Heart Rate: Systematic Review. *JMIR Mhealth Uhealth*. **8** (9), e18694 (2020). 32897239 10.2196/18694 PMC7509623

[20] Scheid, J. L., Reed, J. L. & West, S. L. Commentary: Is Wearable Fitness Technology a Medically Approved Device? Yes and No. *Int. J. Environ. Res. Public. Health.* **20**(13). (2023). 10.3390/ijerph20136230 PMC10341580 37444078

[21] Kasiak, P. S. et al. Validity of the Maximal Heart Rate Prediction Models among Runners and Cyclists. *J. Clin. Med.* **12**(8), 2884 (2023). 10.3390/jcm12082884 PMC10146295 37109218

[22] Kaufmann, S., Gronwald, T., Herold, F. & Hoos, O. Heart Rate Variability-Derived Thresholds for Exercise Intensity Prescription in Endurance Sports: A Systematic Review of Interrelations and Agreement with Different Ventilatory and Blood Lactate Thresholds. *Sports Med. Open.* **9** (1), 59 (2023). 37462761 10.1186/s40798-023-00607-2 PMC10354346

[23] Rogers, B., Giles, D., Draper, N., Hoos, O. & Gronwald, T. A New Detection Method Defining the Aerobic Threshold for Endurance Exercise and Training Prescription Based on Fractal Correlation Properties of Heart Rate Variability. *Front. Physiol.* **11**, 596567 (2020). 33519504 10.3389/fphys.2020.596567 PMC7845545

[24] Rogers, B., Giles, D., Draper, N., Mourot, L. & Gronwald, T. Detection of the Anaerobic Threshold in Endurance Sports: Validation of a New Method Using Correlation Properties of Heart Rate Variability. *J. Funct. Morphology Kinesiol.* **6** (2), 38 (2021). 10.3390/jfmk6020038 PMC8167649 33925974

[25] Tanner, V., Millet, G. P. & Bourdillon, N. Agreement Between Heart Rate Variability - Derived vs. Ventilatory and Lactate Thresholds: A Systematic Review with Meta-Analyses. *Sports Med. Open.* **10** (1), 109 (2024). 39379776 10.1186/s40798-024-00768-8 PMC11461412

[26] Tanner, R. E. et al. Age-related differences in lean mass, protein synthesis and skeletal muscle markers of proteolysis after bed rest and exercise rehabilitation. *J. Physiol.* **593** (18), 4259–4273 (2015). 26173027 10.1113/JP270699 PMC4594296

[27] Sempere-Ruiz, N., Sarabia, J. M., Baladzhaeva, S. & Moya-Ramón, M. Reliability and validity of a non-linear index of heart rate variability to determine intensity thresholds. *Front. Physiol. ***15**, 1329360 (2024). 10.3389/fphys.2024.1329360 PMC10875128 38375458

[28] Gronwald, T. & Hoos, O. Correlation properties of heart rate variability during endurance exercise: A systematic review. *Ann. Noninvasive Electrocardiol.* **25** (1), e12697 (2020). 31498541 10.1111/anec.12697 PMC7358842

[29] Herzig, D., Asatryan, B., Brugger, N., Eser, P. & Wilhelm, M. The Association Between Endurance Training and Heart Rate Variability: The Confounding Role of Heart Rate. *Front. Physiol.* **9**, 2018 (2018). 10.3389/fphys.2018.00756 PMC6018465 29971016

[30] Eclache, J. P., Garcia-Tabar, I. & Gorostiaga, E. M. A new objective method for determining exercise gas exchange thresholds by respiratory frequency in middle-aged men. *Eur. J. Appl. Physiol.* **124** (11), 3227–3240 (2024). 38849689 10.1007/s00421-024-05520-4 PMC11519234

[31] Contreras-Briceño, F. et al. Estimation of ventilatory thresholds during exercise using respiratory wearable sensors. *npj Digit. Med.* **7** (1), 198 (2024). 39060511 10.1038/s41746-024-01191-9 PMC11282229

[32] Roberts, J. D. Jr., Walton, R. D., Loyer, V., Bernus, O. & Kulkarni, K. Open-source software for respiratory rate estimation using single-lead electrocardiograms. *Sci. Rep.* **14** (1), 167 (2024). 38168512 10.1038/s41598-023-50470-0 PMC10762020

[33] Charlton, P. H. et al. Breathing Rate Estimation From the Electrocardiogram and Photoplethysmogram: A Review. *IEEE Rev. Biomed. Eng.* **11**, 2–20 (2018). 29990026 10.1109/RBME.2017.2763681 PMC7612521

[34] Anosov, O., Patzak, A., Kononovich, Y. & Persson, P. B. High-frequency oscillations of the heart rate during ramp load reflect the human anaerobic threshold. *Eur. J. Appl. Physiol.* **83** (4–5), 388–394 (2000). 11138580 10.1007/s004210000302

[35] Nabetani, T., Ueda, T. & Teramoto, K. Measurement of ventilatory threshold by respiratory frequency. *Percept. Mot Skills*. **94** (3 Pt 1), 851–859 (2002). 12081290 10.2466/pms.2002.94.3.851

[36] Garner, K. K., Pomeroy, W. & Arnold, J. J. Exercise Stress Testing: Indications and Common Questions. *Am. Fam Physician*. **96** (5), 293–299 (2017). 28925651

[37] Raa, A. et al. Validation of a point-of-care capillary lactate measuring device (Lactate Pro 2). *Scand. J. Trauma. Resusc. Emerg. Med.* **28** (1), 83 (2020). 32811544 10.1186/s13049-020-00776-z PMC7437027

[38] Cheng, B. et al. A new approach for the determination of ventilatory and lactate thresholds. *Int. J. Sports Med.* **13** (7), 518–522 (1992). 1459746 10.1055/s-2007-1021309

[39] Shookster, D., Lindsey, B., Cortes, N. & Martin, J. R. Accuracy of Commonly Used Age-Predicted Maximal Heart Rate Equations. *Int. J. Exerc. Sci.* **13** (7), 1242–1250 (2020). 33042384 10.70252/XFSJ6815 PMC7523886

[40] Goodyear, M. D. E., Krleza-Jeric, K. & Lemmens, T. The Declaration of Helsinki. *BMJ* **335** (7621), 624–625 (2007). 17901471 10.1136/bmj.39339.610000.BE PMC1995496

[41] Allen, S. E. & Holm, J. L. Lactate: physiology and clinical utility. *J. Veterinary Emerg. Crit. Care*. **18** (2), 123–132 (2008).

[42] Prud’Homme, D. et al. Reliability of assessments of ventilatory thresholds. *J. Sports Sci.* **2** (1), 13–24 (1984).

[43] Milani, J., Milani, M., Cipriano, G. F. B., Hansen, D. & Cipriano Junior, G. Exercise intensity domains determined by heart rate at the ventilatory thresholds in patients with cardiovascular disease: new insights and comparisons to cardiovascular rehabilitation prescription recommendations. *BMJ Open. Sport Exerc. Med.* **9** (3), e001601 (2023). 37533593 10.1136/bmjsem-2023-001601 PMC10391816

[44] Milani, J. et al. Accurate prediction equations for ventilatory thresholds in cardiometabolic disease when gas exchange analysis is unavailable: development and validation. *Eur. J. Prev. Cardiol.* **31** (16), 1914–1924 (2024). 38636093 10.1093/eurjpc/zwae149

[45] Iannetta, D. et al. A Critical Evaluation of Current Methods for Exercise Prescription in Women and Men. *Med. Sci. Sports Exerc.* **52** (2), 466–473 (2020). 31479001 10.1249/MSS.0000000000002147

[46] Stergiopoulos, D. C., Kounalakis, S. N., Miliotis, P. G. & Geladas, N. D. Second Ventilatory Threshold Assessed by Heart Rate Variability in a Multiple Shuttle Run Test. *Int. J. Sports Med.* **42** (1), 48–55 (2021). 32770536 10.1055/a-1214-6309

[47] Katsumata, Y. et al. Sweat lactate sensor for detecting anaerobic threshold in heart failure: a prospective clinical trial (LacS-001). *Sci. Rep.* **14** (1), 18985 (2024). 39152287 10.1038/s41598-024-70001-9 PMC11329511

[48] Yang, G., Hong, J. & Park, S. B. Wearable device for continuous sweat lactate monitoring in sports: a narrative review. *Front. Physiol.* **15**, 1376801 (2024). 38638276 10.3389/fphys.2024.1376801 PMC11025537

[49] Van Hoovels, K. et al. Can Wearable Sweat Lactate Sensors Contribute to Sports Physiology? *ACS Sens.* **6** (10), 3496–3508 (2021). 34549938 10.1021/acssensors.1c01403 PMC8546758

[50] Staes, M., Gyselinck, I., Goetschalckx, K., Troosters, T. & Janssens, W. Identifying limitations to exercise with incremental cardiopulmonary exercise testing: a scoping review. *Eur. Respir Rev.* **33**, 173 (2024). 10.1183/16000617.0010-2024 PMC11372471 39231595

[51] Franssen, R. F. W. et al. Inter-observer agreement of preoperative cardiopulmonary exercise test interpretation in major abdominal surgery. *BMC Anesthesiol*. **22** (1), 131 (2022). 35490221 10.1186/s12871-022-01680-y PMC9055752

[52] Kaczmarek, S. et al. Interobserver variability of ventilatory anaerobic threshold in asymptomatic volunteers. *Multidiscip Respir Med.* **14**, 20 (2019). 31198557 10.1186/s40248-019-0183-6 PMC6556958

[53] Neder, J. A. Cardiopulmonary exercise testing applied to respiratory medicine: Myths and facts. *Respir Med.* **214**, 107249 (2023). 37100256 10.1016/j.rmed.2023.107249

[54] Jamnick, N. A., Pettitt, R. W., Granata, C., Pyne, D. B. & Bishop, D. J. An Examination and Critique of Current Methods to Determine Exercise Intensity. *Sports Med.* **50** (10), 1729–1756 (2020). 32729096 10.1007/s40279-020-01322-8

[55] Balady, G. J. et al. Clinician’s Guide to Cardiopulmonary Exercise Testing in Adults. *Circulation* **122** (2), 191–225 (2010). 20585013 10.1161/CIR.0b013e3181e52e69

[56] ATS/ACCP Statement on cardiopulmonary exercise testing. *Am. J. Respir Crit. Care Med.* ;**167**(2):211–277. (2003). 12524257 10.1164/rccm.167.2.211

[57] Gilgen-Ammann, R., Schweizer, T. & Wyss, T. RR interval signal quality of a heart rate monitor and an ECG Holter at rest and during exercise. *Eur. J. Appl. Physiol.* **119** (7), 1525–1532 (2019). 31004219 10.1007/s00421-019-04142-5

